# Prevalence and morphology of the gastrocnemius tertius: anatomical study and literature review

**DOI:** 10.1007/s00276-025-03625-9

**Published:** 2025-03-28

**Authors:** George Triantafyllou, Nicol Zielinska, Maria Piagkou, Krzysztof Koptas, Andrzej Węgiel, Łukasz Olewnik

**Affiliations:** 1 Department of Anatomy, School of Medicine, Faculty of Health Sciences, National and Kapodistrian University of Athens, 75 Mikras Asias str., Goudi, 11527 Athens, Greece; 2 Department of Clinical Anatomy, Masovian Academy in Płock, Plock, Poland; 3 Department of Anatomical Dissection and Donation, Medical University of Lodz, Lodz, Poland

**Keywords:** Gastrocnemius muscle, Variation, Gastrocnemius tertius, Entrapment, Compression, Popliteal artery compression, Tibial nerve entrapment

## Abstract

**Background:**

The gastrocnemius muscle (GM) third head or gastrocnemius tertius (GT) is a well-described GM variant. The purpose of the current study was to investigate the GT prevalence and morphology (including the proximal and distal attachments, and relationship with the neurovascular structures) in a cadaveric Central European population.

**Methods:**

A total of seventy-three lower limbs were dissected and investigated for GT presence; when the variant was identified, morphometric measurements were obtained.

**Results:**

The GM third head was observed in 10.96% (8/73 cases) under the form of two different morphological variants. The commonest variant type (6 cases) with proximal attachment from the femur posterior surface and fused with the GM lateral head. The second variant type (2 cases) proximal attachment was from the femur posterior surface and fused with the GM medial head. To understand this variation, we performed a brief literature review with meta-analysis. The GT variant has been identified with a pooled prevalence of 4.34%, under great morphological variability.

**Conclusion:**

Clinicians, especially orthopaedics, should be aware of this variant, as it has been proven to cause popliteal neurovascular compression.

## Introduction

The superficial muscles of the posterior leg compartment: the gastrocnemius muscle (GM), the soleus muscle (SM), and the plantaris muscle (PM) form the calcaneal (of Achilles) tendon and insert into the calcaneus. The two-headed GM consists of a lateral and medial head (LH and MH) originating from the lateral and medial femoral epicondyle [6]. Both heads are inserted by a strong tendon into the posterior surface of the calcaneus [6]. The GM and the SM determine the plantar flexion of the foot and its supination and help with the knee flexion [8]. A well-described GM variant is the existence of a third head, the so-called gastrocnemius tertius (GT) (of Kelch) or caput tertium [15]. The GM third head is the most common variant of the muscle [6]. It may arise from the femur popliteal surface, lateral epicondyle, knee joint capsule, or even the biceps femoris long head [28]. It usually joins the MH (Bergman et al. 1995). The GT has an overall frequency of 2.9–5.5% [28]. Frey [11] classified the GT into 12 types based on their origin and insertion. The GT variant morphology has clinical implications, since it may cross the popliteal neurovascular structures and has been associated with entrapment syndromes [11, 28].

The current cadaveric study investigates the GT incidence, proximal and distal attachments, and relationship with the popliteal neurovascular structures. Therefore, the purpose of the study was to assess the GT possible presence and, when identified, its morphological variability. The developmental background, the prevalence in different populations with a narrative review, and possible clinical significance are further discussed.

## Materials and methods

A total of 73 adult formalin-fixed lower limbs (36 on the left, and 37 on the right side) were examined to determine the GM morphological variants, particularly the GT presence. The sample was derived from the Department of Anatomical Dissection and Donation (Medical University of Lodz, Central European Population). Dissection started in the legs, removing the skin and superficial fascia, to expose the GM. A meticulous dissection of the popliteal fossa was also performed. After dissection, the following GM morphological characteristics were assessed:

- The GT presence (proximal attachment, course, and distal attachment),
- The GT morphometric details (muscle belly length, thickness, and lengths of the muscle’s proximal attachment and distal attachment), and
- The GT relationship with popliteal neurovascular structures.

An electronic digital caliper was used for all measurements (*Mitutoyo Corporation, Kawasaki-shi, Kanagawa, Japan*).

A scoping literature review was also performed to identify the studies reporting the GT prevalence. It was performed through the online databases *PubMed* and *Google Scholar.* Then, a brief meta-analysis was conducted to calculate the GT presence pooled prevalence through the R programming software.

## Results

The GM was identified in all the lower limbs (73 in total). Among them, the GT was found in 8 (4 left and 4 right) out of 73 specimens (10.96%). Concerning laterality, the GT was bilaterally identified in 3 (2 female and 1 male) cadavers, and unilaterally in 2 (1 female and 1 male) cadavers.

**Fig. 1:**
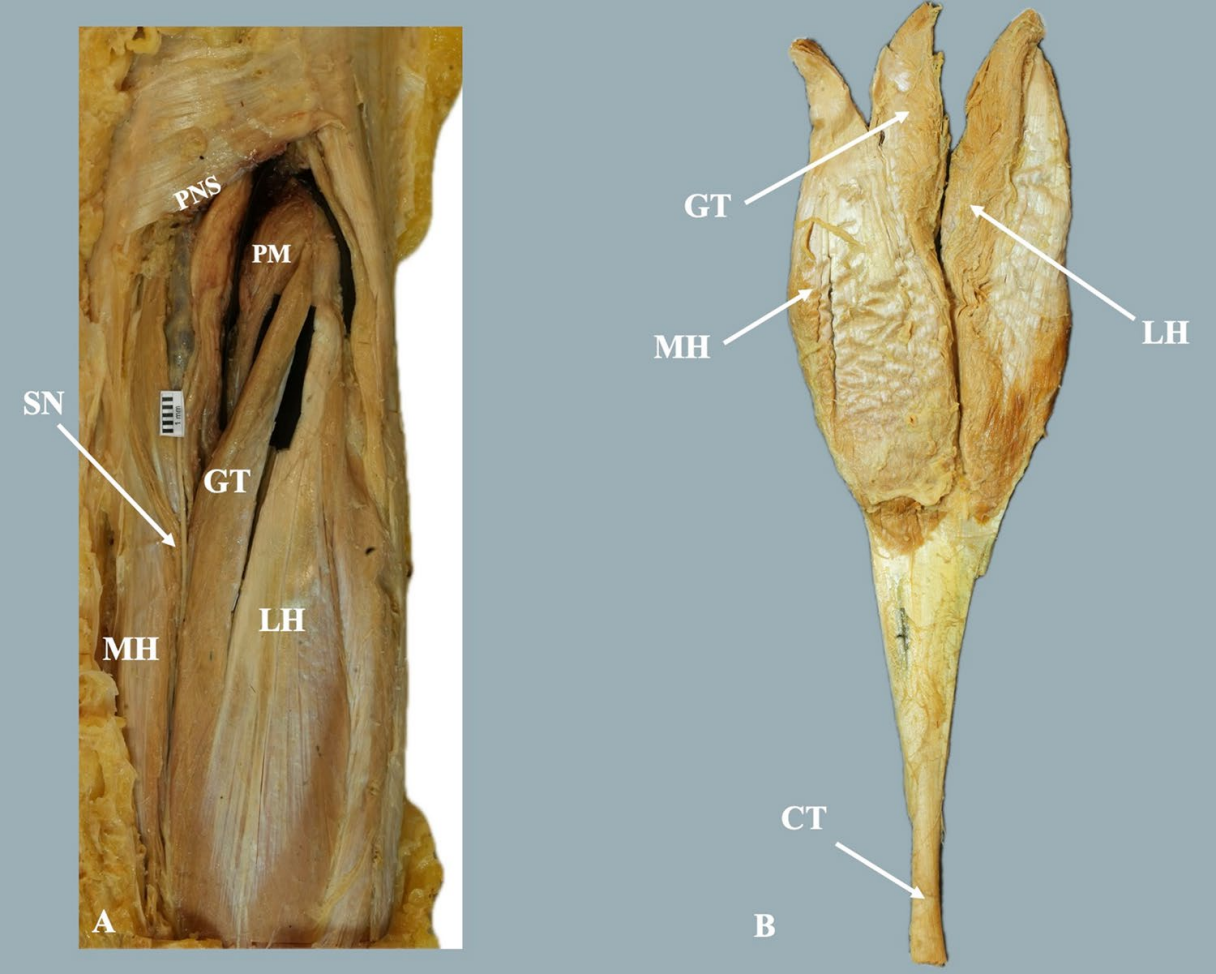
**A** Dissection of the gastrocnemius tertius (GT, third head) variant fused with the gastrocnemius muscle (GM) lateral head (LH). MH- medial head, PNS- popliteal neurovascular structures, SN- sural nerve. **B** Dissection and removal of the gastrocnemius muscle (GM) and the identification of the gastrocnemius tertius (GT) fused with the GM medial head (MH)

**Fig. 2:**
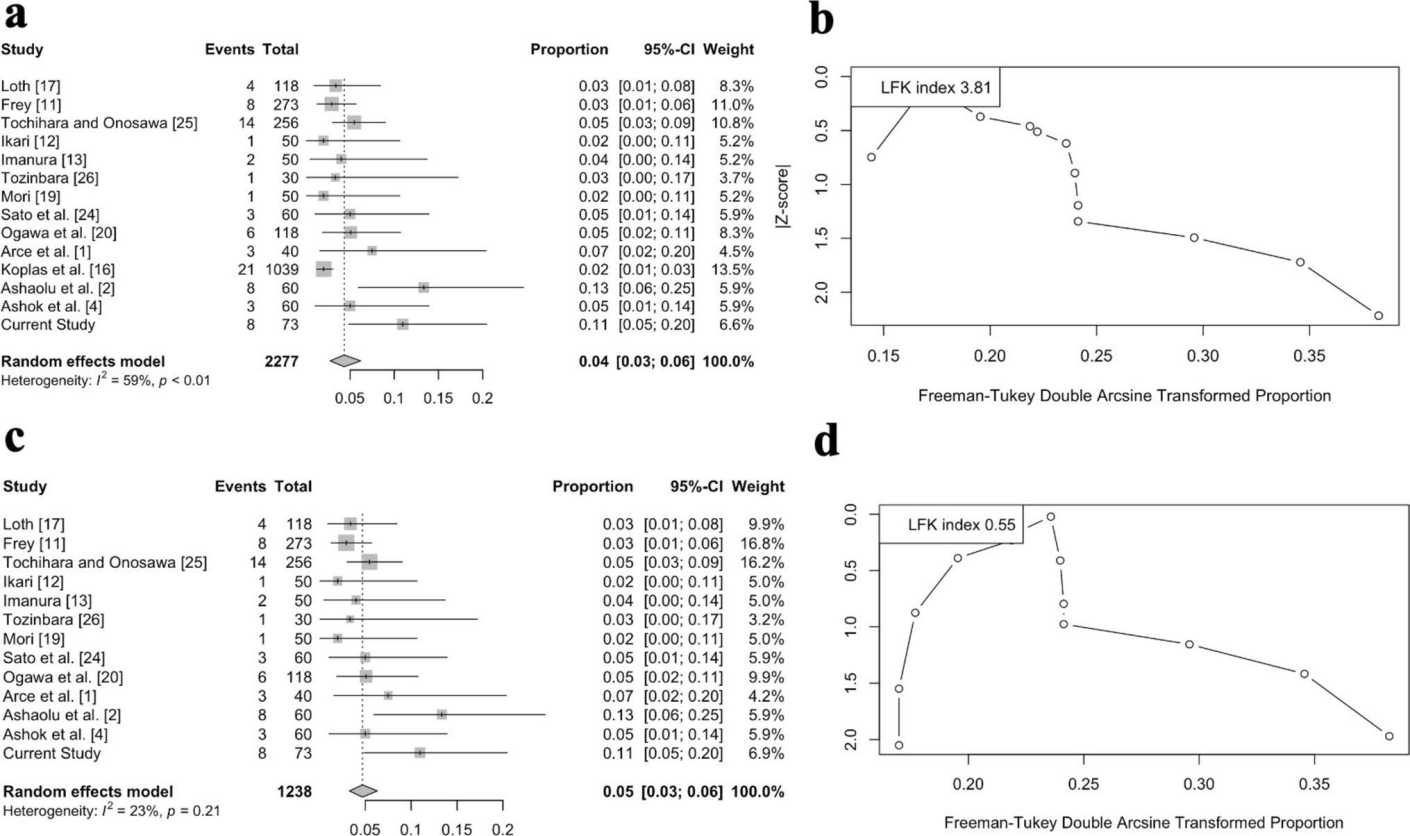
The Forest and DOI plots for the gastrocnemius tertius pooled prevalence. **a**, **b** With all studies included **c**, **d** Without Koplas et al. [16] imaging study

In 6 cases (one female and male cadaver bilaterally, and two male cadavers unilaterally), the GT proximal attachment was from the posterior distal surface of the femur, near the midline, and was fused with the GMLH. In these cases, the popliteal neurovascular structures were located medially to the GM third head, while the tibial nerve (TN) provided innervation to the third head (Fig. 1). The presence of the GT adjacent to the GMLH forces the location of the PM posteriorly to the GM and GT. In 2 cases (one female cadaver bilaterally), the GT proximal attachment was from the femur posterior distal surface, close to the midline, and was fused with the GMMH. In these cases, the popliteal neurovascular structures were located laterally to the third head, and the TN supplied the GT (Fig. 2). The GT morphometric measurements are summarized in Table 1.

**Table 1:** Morphometric parameters of gastrocnemius tertius (GT)

Morphometric parameters	Minimum value (mm)	Mean value (mm)	Maximum value (mm)
PA Length	7.04	11.29	14.17
PA Thickness	1.83	5.74	8.06
Muscle belly Length	63.15	84.58	99.92
DA Length	12.38	13.34	14.61
DA Thickness	4.80	6.45	9.03

The minimum, maximum (range), and mean values are presented. All parameters are expressed in mm. *PA* proximal attachment, *DA* distal attachment. The width was measured as the lateromedial distance, the thickness was expressed as an anteroposterior distance and length as the craniocaudal distance from the uppermost point of origin to the lowermost insertion point

Fourteen studies (Table 2) reporting the GT prevalence and morphology were retrieved, including the current one. Based on the total number of articles (k = 14) and the total sample (n = 2277 lower limbs), the GT pooled prevalence was estimated at 4.34% (95% CI 2.75–6.21). The Higgins I^2^ was calculated at 59.5% (moderate heterogeneity). A subgroup analysis for the nationality was also performed and it did not depict significant difference (*p* = 0.1282). A DOI plot was conducted to assess possible small-study effect, and the LFK index was + 3.81 (major asymmetry). Then, we removed the study by Koplas et al. [16] due to its significant higher sample compared to the cadaveric studies. The pooled prevalence, without this study, was observed at 4.69% (95% CI 3.27–6.32), with Higgins I^2^ at 22.9% (not necessary heterogeneity) and the DOI plot with LFK index (+ 0.55) retrieved no small-study effect. The statistical meta-analysis is summarized in Fig. 2.

**Table 2:** The incidence of the gastrocnemius tertius (GT), among different populations

References	Year	Origin	Type of study	Sample (n =)	GT presence (n =)
Loth [17]	1912	German	Cadaveric	118	4
Frey [11]	1919	German	Cadaveric	273	8
Tochihara and Onosawa [25]	1932	Japanese	Cadaveric	256	14
Ikari [12]	1945	Japanese	Cadaveric	50	1
Imanura [13]	1949	Japanese	Cadaveric	50	2
Tozinbara [26]	1960	Japanese	Cadaveric	30	1
Mori [19]	1964	Japanese	Cadaveric	50	1
Sato et al. [24]	1985	Japanese	Cadaveric	60	3
Ogawa et al. [20]	2005	Japanese	Cadaveric	118	6
Arce et al. [1]	2008	Argentinian	Cadaveric	40	3
Koplas et al. [16]	2009	American	MRI	1.039	21
Ashaolu et al. [2]	2014	Nigerian	Cadaveric	60	8
Ashok et al. [4]	2017	Indian	Cadaveric	60	3
Current study	2024	Polish	Cadaveric	73	8

## Discussion

### Embryological background and comparative anatomy of the gastrocnemius tertius (GT)

Generally, the lateral portion of the flexor plate of the leg gives rise to GM and SM [5]. GM anlage is more lateral and superficial of the two muscles and shows two incompletely separated heads [5]. In an 11 mm long embryo, near the knee, a mass of slightly differentiated tissue lying superficial to the TN represents the GM-SM group of muscles [5]. In a 14 mm long embryo, the GM group is connected by a mass of tissue with the calcaneus blastema [5]. Nevertheless, in a 20 mm long embryo, the GM and SM have begun to extend “tibial wards” over the TN, the calcaneus tendon is differentiated, while the LH has formed a tendinous attachment above the femur lateral epicondyle, but the MH has not finally formed [5]. During the 2nd developmental month, the GM heads rapidly developed [5]. Additionally, it is important to investigate the comparative anatomy of those muscles. It seems that the GM-SM group is not homologous in the amphibia, reptiles, and mammals, although there are obvious similarities [18]. In mammals, McMurrich [18] considered the GM MH to be a distinct muscle from the GMLH. The muscle’s ontogeny in man indicates that the LH and MH of the GM derive from an anlage located on the fibular side of the leg [5].

### Morphological variability of the gastrocnemius tertius (GT)

In the current study, the GT was identified in 10.96% (8/73 lower limbs) compared to Bergman’s Comprehensive Encyclopedia of Anatomic Variations reporting prevalence between 2.9 and 5.5% [5]. Nevertheless, our meta-analysis retrieved a GT pooled prevalence was estimated at 4.34%. Therefore, the higher prevalence reported in the current study could be attributed to the nationality of the specimens (Central European population).

In the current study, the GT was identified in 6 cases bilaterally and in 2 cases unilaterally. Koplas et al. [16] in a retrospective magnetic resonance imaging (MRI) study on 1.039 lower limbs, identified the GT in 2% (21 cases, 19 cases unilaterally, and 1 case bilaterally). Few case studies of GT's bilateral existence have been reported by Yildirim et al. [30], Ishii et al. [14] and Tsakotos et al. [27].

In the current study, 6 out of 8 third heads were identified as fusing with the GMLH, following the Koplas et al. [16] study. Contrariwise, Bergman et al. [7] supported that most commonly GT fuses with the GMMH. In the present study, two distinct GT morphological types were observed, concerning the proximal attachment of the third head, its fusion, and its distal attachment. The first GT variant (more frequently identified) had a proximal attachment from the femur posterior surface, fused with the LH of GM, and located lateral to the neurovascular structures (8.2%). In these cases, the PM was identified posteriorly to the GM (in a deeper layer) due to the GT's presence. The second GT variant had a proximal attachment from the femur posterior surface, fused with the GMMH, and located medial to the neurovascular structures (2.7%). The TN innervated the GT in all cases (11 lower limbs). Interestingly, Tsakotos et al. [27] identified a case where the GT insertion was located with a separate tendon into the AT, that we did not identify in the current study.

The first and unique classification system for GT's morphological types was presented by Frey [11], who classified the GT variants based on the origin, insertion, and possible compression of the popliteal neurovascular structures. In total, 12 GT morphological variants were identified [11], but this classification was quite complicated with minor differences between types, and it was not followed by researchers. Frey [11] defined the GT origin according to the relationship of the third head with the TN and popliteal vessels and identified its insertion either into the GM LH or MH. A unique type had an origin in the popliteal fossa and was inserted into the Achilles tendon with a distinct tendon. Frey [11] highlighted that GT cases that course over the popliteal neurovascular structures could compress on them.

Other rarer variations of GM can be observed. In the present study, the presence of quadriceps (four-headed) GM or was not observed in the current cadaveric lower limb series (73 cases). Koplas et al. [16] identified one case (1/1039 cases, 0.09%) of a GM with two accessory heads (four-headed GM) in their MRI study. In their case study, Oztoprak et al. [21] identified two accessory heads that arose from both LH and MH, resulting in compression of the popliteal artery (PA) and claudication problems. Interestingly, Ashaolu et al. [2] observed a bilateral quadriceps GM on a cadaveric specimen. Lastly, Koplas et al. [16] highlighted the presence of coexistent variants in the popliteal fossa, such as a popliteal muscle originating from the lateral retinaculum and accessory popliteal muscle.

### Clinical implications of gastrocnemius tertius (GT)

The GT has been implicated in leading to the compression of the popliteal neurovascular bundle [31]. This entrapment may affect one or more components of the popliteal neurovascular bundle. Most commonly the artery is affected; however, the sural nerve (SN) can also be entrapped due to its course between the GM heads. SN entrapment would lead to paresthesia on the posterior compartment of the foot. PA entrapment syndrome is relatively uncommon and often appears in young and healthy patients. Most commonly, it appears with intermittent claudication and pain during exercise [9]. Clinical cases have been reported with PA entrapment by GM variants, such as in GT presence [29]. Nerve entrapment symptoms appear as tibial or sciatic neuropathy, leading to GM atrophy [10, 16]. In rare instances, common peroneal neuropathy may also appear. Additionally, compression of the popliteal vessels can result in either a genuine thrombophlebitic or a pseudothrombophlebitic syndrome. The potential diagnoses for syndromes linked to the neurovascular bundle compression encompass various clinical entities such as intraneural ganglion cyst affecting the sciatic nerve lower divisions, adventitial cyst in the PA, thrombophlebitic syndrome due to lower extremity deep vein thrombosis, synovial sarcoma in the knee joint, popliteal entrapment syndrome, posterior compartment syndrome in the lower leg, PA aneurysm, and Baker cyst. Among those clinical entities, the GM variants and particularly its additional head orientation should also be kept in mind [10, 16]. Thorough clinical examination of the lower limb targeting the popliteal fossa with ultrasonography imaging [22] and/or magnetic resonance imaging (MRI) of the area, is of paramount importance for confirming proper diagnosis.

### Limitations

The present study has a few limitations. Although the sample was considered adequate (n = 73), the low number of identified cases (n = 8) did not allow us to perform proper statistical analysis. In addition, the sample was limited to a specific population (Poland, Central Europe). Therefore, wider populations and more extensive studies will enhance the current knowledge about the prevalence of the GT variant, and possible identification of its morphological variability and potential of compression of deeply neurovascular structures.

## Conclusions

The GT (GM third head) was identified in 10.96% of the current study, with two different morphological types. The first and most common GT type (75%) fused with the GMLH, and the second type (25%) fused with the GMMH. After analyzing the current literature and conducting a brief meta-analysis, the pooled prevalence of GT was calculated at 4.34%. The GM third head has been implicated in compression of the popliteal neurovascular structures; therefore, clinicians should be aware of this variant.

## Data Availability

No datasets were generated or analysed during the current study.
